# Enniatins A1 and B1 Modulate Calcium Flux through Alternative Pathways beyond Mitochondria

**DOI:** 10.1021/acs.jafc.4c04242

**Published:** 2024-06-20

**Authors:** Nadia Pérez-Fuentes, Rebeca Alvariño, Amparo Alfonso, Jesús González-Jartín, Mercedes R. Vieytes, Luis M. Botana

**Affiliations:** † Departamento de Farmacología, Facultad de Veterinaria, IDIS, Universidad de Santiago de Compostela, Lugo 27002, Spain; ‡ Departamento de Fisiología, Facultad de Veterinaria, IDIS, Universidad de Santiago de Compostela, Lugo 27002, Spain

**Keywords:** enniatin A1, enniatin B1, calcium fluxes, endoplasmic reticulum, mitochondria

## Abstract

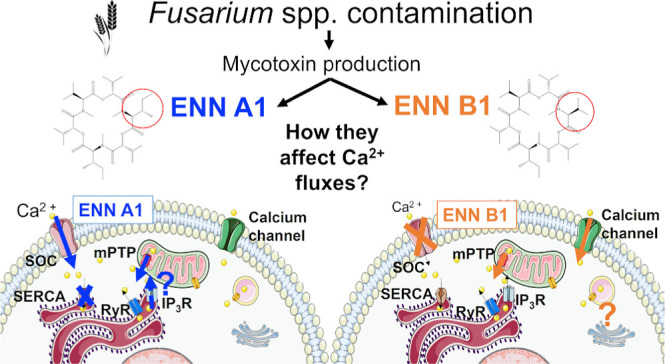

Enniatins (ENNs) A1 and B1, previously considered ionophores, are emerging mycotoxins with effects on Ca^2+^ homeostasis. However, their exact mechanism of action remains unclear. This study investigated how these toxins affect Ca^2+^ flux in SH-SY5Y cells. ENN A1 induced Ca^2+^ influx through store-operated channels (SOC). The mitochondrial uncoupler FCCP reduced this influx, suggesting that the mitochondrial status influences the toxin effect. Conversely, ENN B1 did not affect SOC but acted on another Ca^2+^ channel, as shown when nickel, which directly blocks the Ca^2+^ channel pore, is added. Mitochondrial function also influenced the effects of ENN B1, as treatment with FCCP reduced toxin-induced Ca^2+^ depletion and uptake. In addition, both ENNs altered mitochondrial function by producing the opening of the mitochondrial permeability transition pore. This study describes for the first time that ENN A1 and B1 are not Ca^2+^ ionophores and suggests a different mechanism of action for each toxin.

## 1 Introduction

Enniatins (ENNs) are a group of emerging mycotoxins produced by various fungi species, including *Fusarium*, *Aspergillus*, and *Penicillium* genera. These N-methylated cyclic hexadepsipeptides have over 29 analogues, with enniatin A (ENN A), enniatin A1 (ENN A1), enniatin B (ENN B), and enniatin B1 (ENN B1) being the best known. ENNs contaminate a wide range of food and feed, particularly cereals, milk, and fish, and exhibit diverse biological activities, including antifungal, insecticidal, and antitumor properties. These mycotoxins have shown cytotoxic effects in various cell types, raising concerns about their presence in food.^1−3^ These studies indicate that ENNs A, A1, B, and B1 can reduce cell viability and induce apoptotic death in different cell lines at the micromolar range.^3−5^ In Caco-2 cells, their effects were associated with the generation of reactive oxygen species (ROS) and with the disruption of mitochondrial function by altering the mitochondrial membrane potential (MMP).^5^ In SH-SY5Y cells, ENNs A1 and B1 did not affect MMP, and only ENN A1 increased ROS production.^3,4^

Despite their widespread occurrence and cytotoxicity, the precise mechanism of action of ENNs remains unclear. Traditionally, ENNs have been identified as ionophores since it was proposed in 1973 that ENN B forms complexes on cell membranes, facilitating the passage of ions.^6^ Subsequently, all ENNs were considered as ionophores.^7,8^ However, information on their ionophoric behavior is limited. Some studies suggest that a mixture of ENNs A, A1, B, and B1 may exhibit potassium-selective ionophoric properties.^8^ In addition, the effects of ENNs have been linked to fluctuations in divalent cations, such as calcium. Recently, ENNs A1 and B1 have been shown to alter Ca^2+^ fluxes in SH-SY5Y cells in a dose-dependent manner, and ENNs A and B were found to affect intracellular Ca^2+^ pools.^4^ Furthermore, ENN A increases cytosolic Ca^2+^ levels in erythrocytes and affects store-operated channels (SOC) in human neuroblastoma cells.^9,10^ Regarding ENN B, it decreases mitochondrial Ca^2+^ retention in boar spermatozoa and modifies calcium fluxes through the mitochondrial pathway in SH-SY5Y cells.^8,9^

Ca^2+^ is a crucial element for maintaining cellular homeostasis and plays a vital role at the neuronal level. Its precise regulation is essential for various cellular processes, including neurotransmission, gene expression, and cell survival. Ca^2+^ acts as a second messenger that influences synaptic plasticity and signal transmission.^11,12^ The intricate control of cellular calcium levels involves dynamic interactions between various cellular compartments, which serve as reservoirs. The endoplasmic reticulum (ER) acts as a major intracellular calcium store, releasing the ion into the cytoplasm upon stimulation. It also accumulates the ion in response to specific cellular events, thereby maintaining calcium homeostasis. Mitochondria play a dual role by buffering cytoplasmic calcium and regulating their own calcium levels, which impacts energy production and cell fate. The nucleus, Golgi apparatus, peroxisomes, and endolysosomes also contribute to calcium storage and signaling, collectively orchestrating a finely tuned balance critical for neuronal function.^13^

Given this background, the objective of this study was to investigate the intracellular pools involved in the effects of ENN A1 and ENN B1 on Ca^2+^ homeostasis in SH-Sy5Y human neuroblastoma cells. In a broader context, understanding the impact of ENNs on cellular processes, including Ca^2+^ homeostasis, is crucial for assessing their potential health risks and developing strategies to mitigate their effects.

## 2 Materials and Methods

### 2.1 Reagents and Solutions

Thapsigargin (Tg) was bought from Enzo Life Sciences (Lausen, Swiss). Dulbecco’s modified Eagle’s medium: F-12 nutrient Mix (DMEM/F-12), penicillin–streptomycin (10,000 U/mL), trypsin/EDTA (0.05%), and glutamax were obtained from Thermo Fisher Scientific (Madrid, Spain). ENN A1, ENN B1 (purity >95%), 1-[β-(3-(4-methoxyphenyl)propoxy)-4-methoxyphenethyl]-1H-imidazole·HCl (SKF96365), carbonyl cyanide 4-(trifluoromethoxy) phenylhydrazone (FCCP), nickel chloride hexahydrate (NiCl_2_), and other chemical reagents were purchased from Merck (Madrid, Spain). Stock solutions of ENNs were prepared in DMSO, and serial dilutions were made in a culture medium. The DMSO concentration was maintained under 0.05% in all experiments.

Umbreit solution was composed of (in mM) 119.0 NaCl, 22.85 NaHCO_3_, 5.94 KCl, 1.2 MgSO_4_, 1.2 NaH_2_PO_4_, 0.1% glucose, and 1.0 CaCl_2._ Phosphate-buffered saline (PBS) was composed of (in mM) 137.0 NaCl, 8.2 Na_2_HPO_4_, 1.5 KH_2_PO_4_, and 3.2 KCl (pH 7.4). The composition of Locke’s buffer was (in mM) 154.0 NaCl, 10.0 HEPES, 5.6 KCl, 5.0 glucose, 3.6 NaHCO_3_, 1.3 CaCl_2_, and 1.0 MgCl_2_. The pH was adjusted between 7.2 and 7.4 in all the assays.

### 2.2 Cell Culture and Treatment

SH-SY5Y, human neuroblastoma cells were purchased from the American Type Culture Collection, number CRL2266. Cells were cultured in DMEM/F-12 medium enriched with 10,000 U/mL penicillin–streptomycin, 10% fetal bovine serum, and 1% glutamax in a humidified atmosphere of 5% CO_2_ and 95% air at 37 °C. Cells were dissociated weekly using 0.05% trypsin/EDTA.

### 2.3 Measurement of Cytosolic-Free Calcium

Cells were plated onto 18 mm coverslips at a density of 5 × 10^5^ cells per well and allowed to grow for 24 h. For the assessment of Ca^2+^ levels, SH-SY5Y cells were washed twice with Umbreit solution enriched with 0.1% BSA. Subsequently, the cells were loaded with FURA-2 AM fluorescent staining at a concentration of 0.5 μM for 6.5 min at 37 °C and 300 rpm. Then, cells were rinsed twice with Umbreit solution, and coverslips were positioned in a temperature-controlled chamber (Life Sciences Resources, UK) as previously explained.^4^ The observation of cells was conducted using a Nikon Diaphot 200 inverted microscope equipped with epifluorescence optics (Nikon 40×, immersion UV-fluor objective).

Dilutions of compounds were prepared in an Umbreit solution lacking Ca^2+^ and directly added to the incubation chamber. Throughout the experiments, pH was maintained between 7.2 and 7.4. The cytosolic calcium ratio was determined from images captured by a fluorescence system consisting of an ultrahigh-speed wavelength switcher (Lambda-DG4) for excitation and an optical filter changer (Lambda 10–2) for emission (Sutter Instruments Co., USA). A xenon arc bulb served as the light source, and specific wavelengths were selected using filters. Cells were alternatively excited at 340 and 380 nm, with emission collected at 505 nm. Each experiment was performed three independent times in duplicate, with an average of 40 cells per visual field.

### 2.4 Flow Cytometry Analysis of Mitochondrial Permeability Transition Pore Opening

The effects of ENN A1 and B1 on mitochondrial permeability transition pore (mPTP) were assessed using the MitoProbe transition pore assay kit (Thermo Fisher Scientific), following the manufacturer’s guidelines.^14,15^ Cells were initially seeded in 12-well plates at a density of 1 × 10^6^ cells per well and allowed to grow for 24 h. SH-SY5Y cells were then suspended in Umbreit solution, loaded with 0.01 μM calcein-AM and 0.4 mM CoCl_2_, and treated with CsA at 0.2 μM and used to inhibit mPTP activation. After 15 min of incubation at 37 °C, cells were treated with 2 μM ENN A1 and 5 μM ENN B1 for 10 min at the same temperature. Then, neuroblastoma cells were suspended in commercial PBS (pH 7.2) (Thermo Fisher Scientific), filtered and kept on ice. FCCP at 10 μM was used as a positive control to induce mPTP opening. The fluorescence intensity of calcein was assessed by flow cytometry at excitation and emission wavelengths of 488 and 517 nm, respectively, using the ImageStreamMKII instrument and ISX MKII software (Amnis Corporation, Luminex Corp, Austin, TX, USA). Fluorescence from 10,000 events was quantified using IDEAS Application 6.0 software (Amnis Corporation, Luminex Corp).

### 2.5 Statical Analysis

The data are expressed as mean ± SEM of three independent experiments. Differences were assessed by a one-way ANOVA followed by Dunnett’s or Tukey’s post hoc tests. Statistical significance was set at *p < 0.05, **p < 0.01, and ***p < 0.001.

## 3 Results

Based on previous work from our group, we extended our investigation into the effects of ENN A1 and ENN B1 on Ca^2+^ fluxes.^4,9^ We have previously analyzed dose–response effects of these toxins and described their ability to deplete cellular Ca^2+^ stores and to induce an influx of this ion in a dose-dependent manner.^4^ We have also investigated the behavior of ENN A1 in the presence of Tg, an inhibitor of the sarcoendoplasmic reticulum Ca^2+^-ATPase (SERCA). Tg depletes Ca^2+^ from the ER, triggering the entry of the ions through the SOC. ENN A1 was found to have a calcium profile similar to Tg, suggesting that it may act on the same organelle as the SERCA inhibitor.^4^

The first step in this study was to determine the effect of ENN B1 on calcium fluxes in the presence of Tg in order to elucidate its interaction with the cellular calcium regulation system. Cells were sequentially treated with 2 μM Tg and 5 μM ENN B1, concentrations that had previously shown pronounced effects on calcium fluxes in this cell line.^4,16^

The percentage of variation in the cytosolic calcium was calculated across different segments. The percentage of reduction or increase in calcium release from reservoirs and calcium uptake was calculated by comparing the effects of the compounds alone to the effects of the compound mixture. In all cases, the signal of the control cells in the corresponding interval was subtracted.

**Figure 1:**
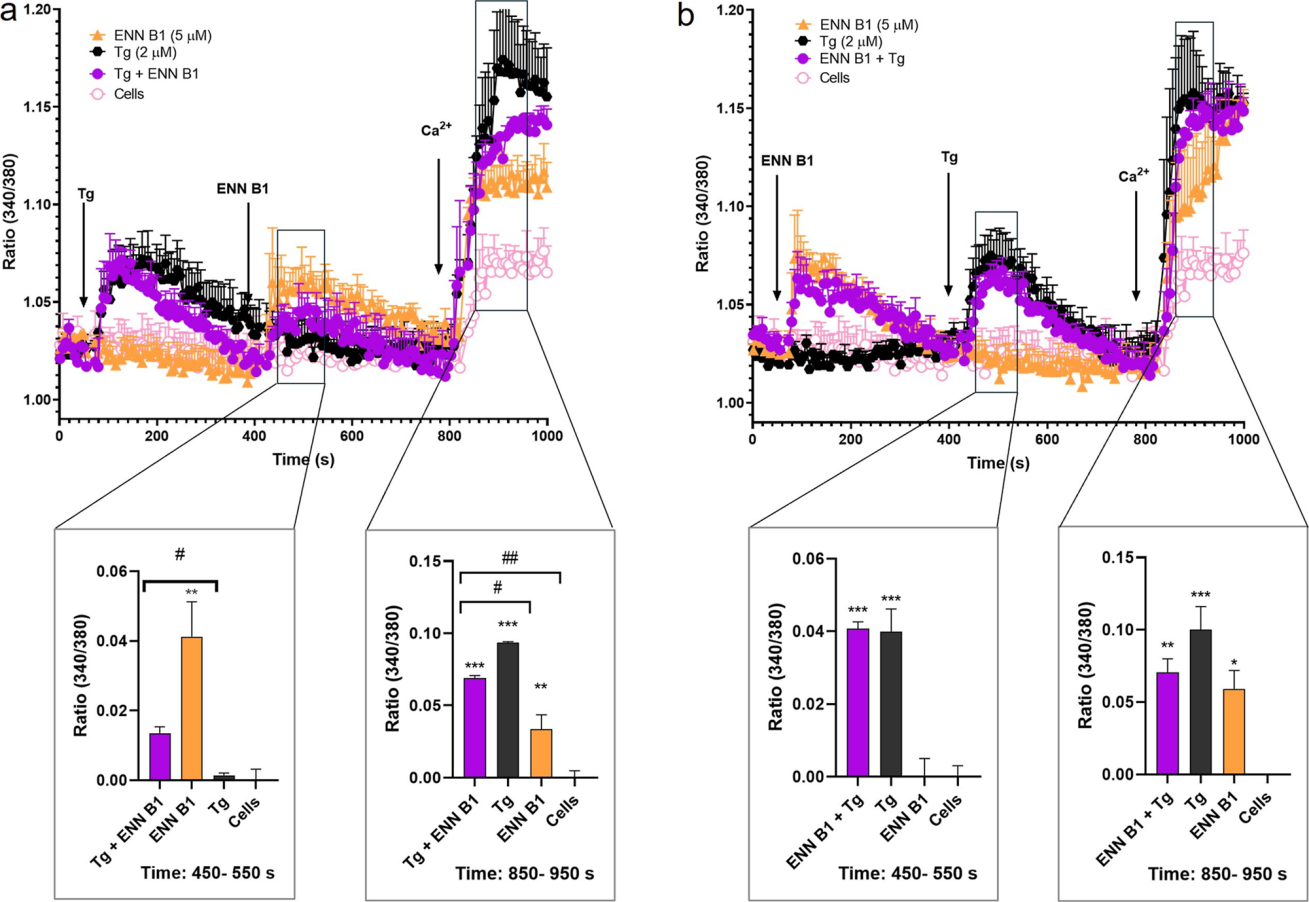
Effects of ENN B1 and Tg on the cytosolic Ca^2+^ profile on SH-SY5Y cells. (a) Cytosolic Ca^2+^ profiles of SH-SY5Y cells pretreated with 2 μM Tg, followed by the addition of 5 μM ENN B1. The left panel shows the quantification of Ca^2+^ release from intracellular pools between the seconds 450 and 550. The right panel presents the quantification of the Ca^2+^ plateau at the time interval 850–950 s. (b) Cytosolic Ca^2+^ profiles of cells preincubated with 5 μM ENN B1, followed by treatment with 2 μM Tg. The left panel presents the quantification of the Ca^2+^ peak (interval 450–550 s), and the right panel shows the quantification of the Ca^2+^ plateau (from 850 to 950 s). Arrows indicate the addition of compounds and 1 mM Ca^2+^ to the bath solution. Statistical significance was determined by one-way ANOVA and Dunnett’s test (***p ≤ 0.001, **p ≤ 0.01, and *p ≤ 0.05 compared to control cells; ###*p* ≤ 0.001, #*p* ≤ 0.05, pairwise comparison). The mean ± SEM of three experiments performed in duplicate.

After pretreatment with Tg, the effects of ENN B1 on intracellular Ca^2+^ pools were reduced by 56.9%, and the Tg-induced Ca^2+^ influx was also diminished by 16.2% (Figure 1a). Then, cells were pretreated with the toxin, followed by the addition of Tg. In this case, there was no significant effect on Tg-induced Ca^2+^-store depletion or Ca^2+^ influx, nor on the maximum peak of Ca^2+^ produced (Figure 1b).

**Figure 2:**
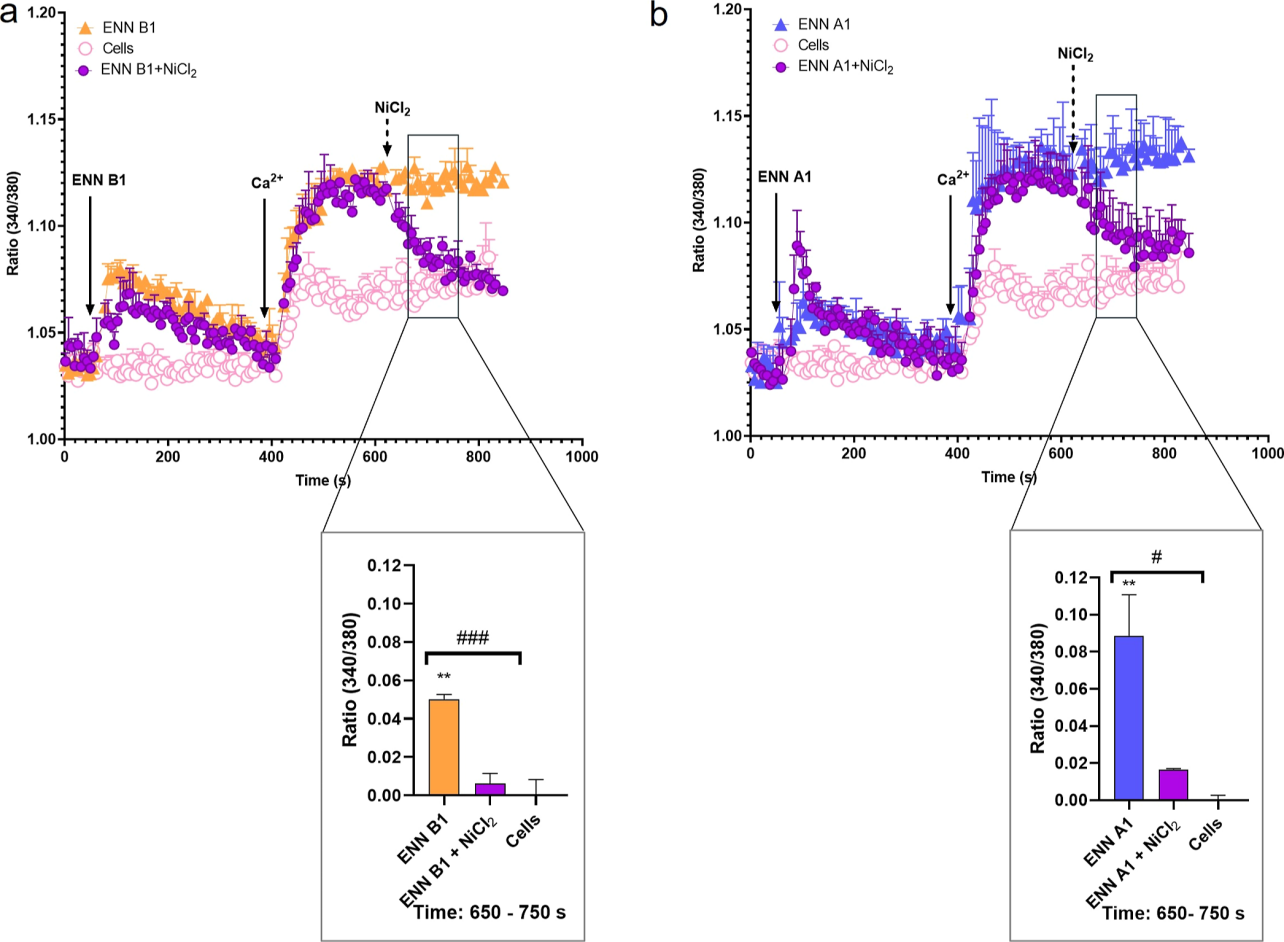
Effect of ENNs B1 and A1 and NiCl_2_ on the cytosolic Ca^2+^ profile of neuroblastoma cells. (a) Cytosolic Ca^2+^ profile of ENN B1-treated cells (solid triangles), cells incubated with 5 μM ENN B1, followed by the addition of 1 mM NiCl_2_ (solid circles), and untreated control cells (open circles). The bottom panel represents the Ca^2+^ quantification at the interval of 650–750 s. (b) Cytosolic Ca^2+^ profile of ENN A1-treated cells (solid triangles), cells incubated with 2 μM ENN A1, followed by the addition of 1 mM NiCl_2_ (solid circles), and untreated control cells (open circles). The bottom panel represents the quantification of Ca^2+^ values from 650 to 750 s of the experiment. Arrows indicate the addition of compounds and 1 mM Ca^2+^ to the bath medium. Statistical significance was determined by one-way ANOVA and Dunnett’s test (***p ≤ 0.001, *p ≤ 0.05 compared to control cells; ###*p* ≤ 0.001, #*p* ≤ 0.05, pairwise comparison). The mean ± SEM of three experiments performed in duplicate.

Since both ENN A1 and ENN B1 resulted in an increase in Ca^2+^ uptake, NiCl_2_, a Ca^2+^ channel blocker that inhibits Ca^2+^ channels by a direct blockade of the pore, was employed at a nontoxic concentration to determine whether the effects of ENN B1 and A1 on Ca^2+^ influx were mediated by cell membrane channels, in order to discard that the toxins were acting as ionophores.^17−20^ The addition of 1 mM of NiCl_2_ after ENN B1 significantly decreased the calcium entry produced by the toxin around 47.5%, achieving almost complete block, as observed in the interval between 650 and 750 s. These results confirmed that the Ca^2+^ uptake induced by the toxin was via a Ca^2+^ channel in the cell membrane (Figure 2a). A similar effect was observed in the case of ENN A1 (2 μM), in which NiCl_2_ reduced the toxin-induced Ca^2+^ entry by 57.1% (Figure 2b), reaching almost basal levels.

**Figure 3:**
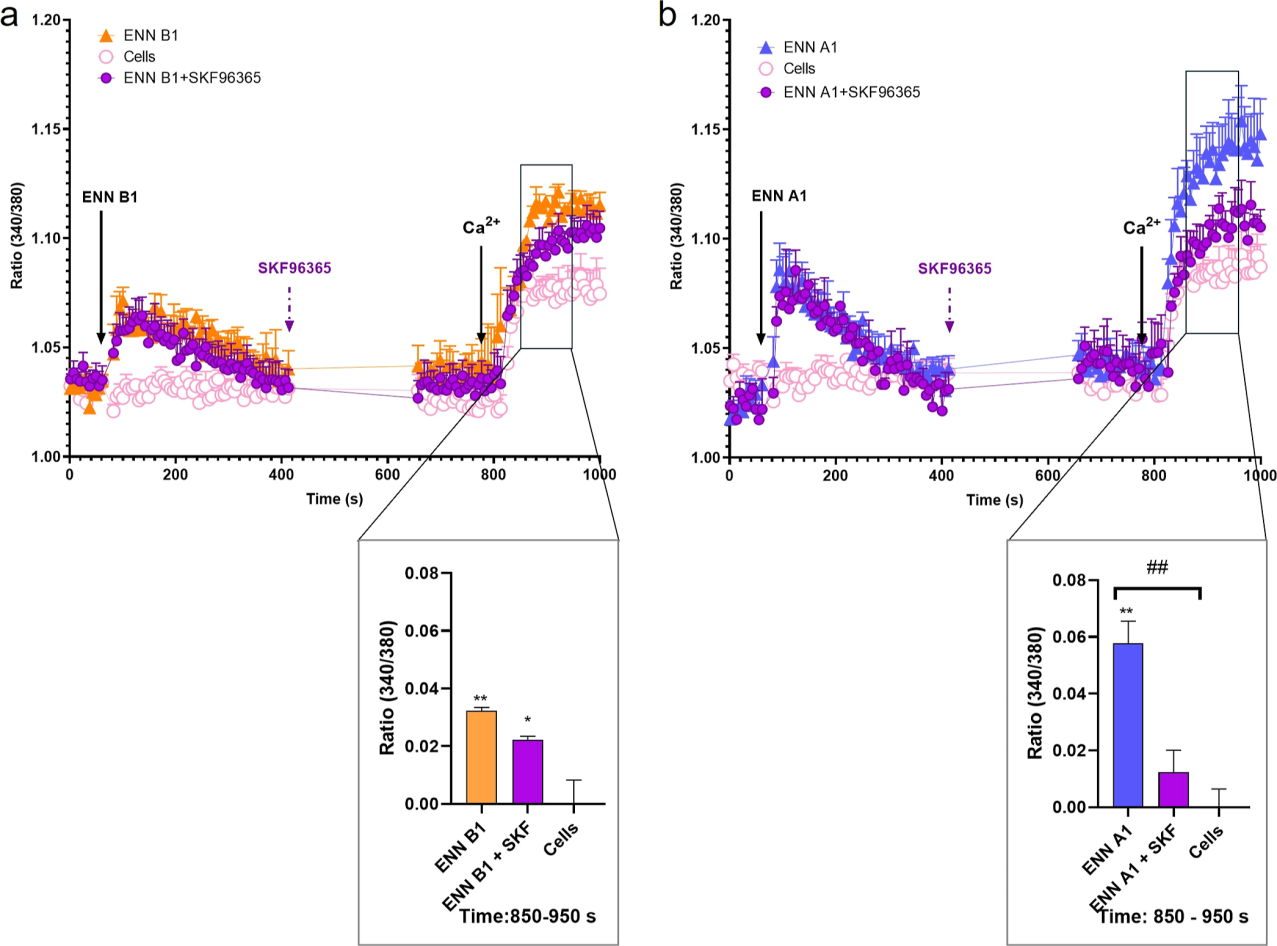
Effect of ENN B1, ENN A1, and SKF96365 on the cytosolic Ca^2+^ profile of neuroblastoma cells. (a) Cytosolic Ca^2+^ profile of ENN B1-treated cells (solid triangles), cells stimulated with 5 μM ENN B1 and incubated for 5 min with 30 μM SKF96365 (solid circles), and untreated control cells (open circles). The panel below represents Ca^2+^ uptake quantification at the region from 850 to 950 s. (b) Cytosolic Ca^2+^ profile of ENN A1-treated cells (solid triangles), cells stimulated with 2 μM ENN A1 and incubated for 5 min with 30 μM SKF96365 (solid circles), and untreated control cells (open circles). The panel below shows quantification of Ca^2+^ uptake at the interval of 850–950 s. Arrows indicate the addition of compounds and 1 mM Ca^2+^ to the bath medium. Statistical significance was determined by one-way ANOVA and Dunnett’s test (***p ≤ 0.001, *p ≤ 0.05 compared to control cells; ###*p* ≤ 0.001, #*p* ≤ 0.05, pairwise comparison). The mean ± SEM of three experiments performed in duplicate.

To follow up with the study, the SOC inhibitor SKF96365 was used to determine whether ENNs A1 and B1 were activating the ion entry through these channels. The effect and concentration of SKF96365 had been previously validated in SH-SY5Y cells.^9^ In the case of ENN B1, preincubation with 30 μM SKF96365 had no effect on the ion influx induced by the toxin, confirming that its effects were mediated by other calcium channels (Figure 3a). By contrast, preincubation with the inhibitor resulted in an almost complete blockade of the Ca^2+^ influx produced by ENN A1, demonstrating its effect on SOC-mediated calcium entry (Figure 3b).

**Figure 4:**
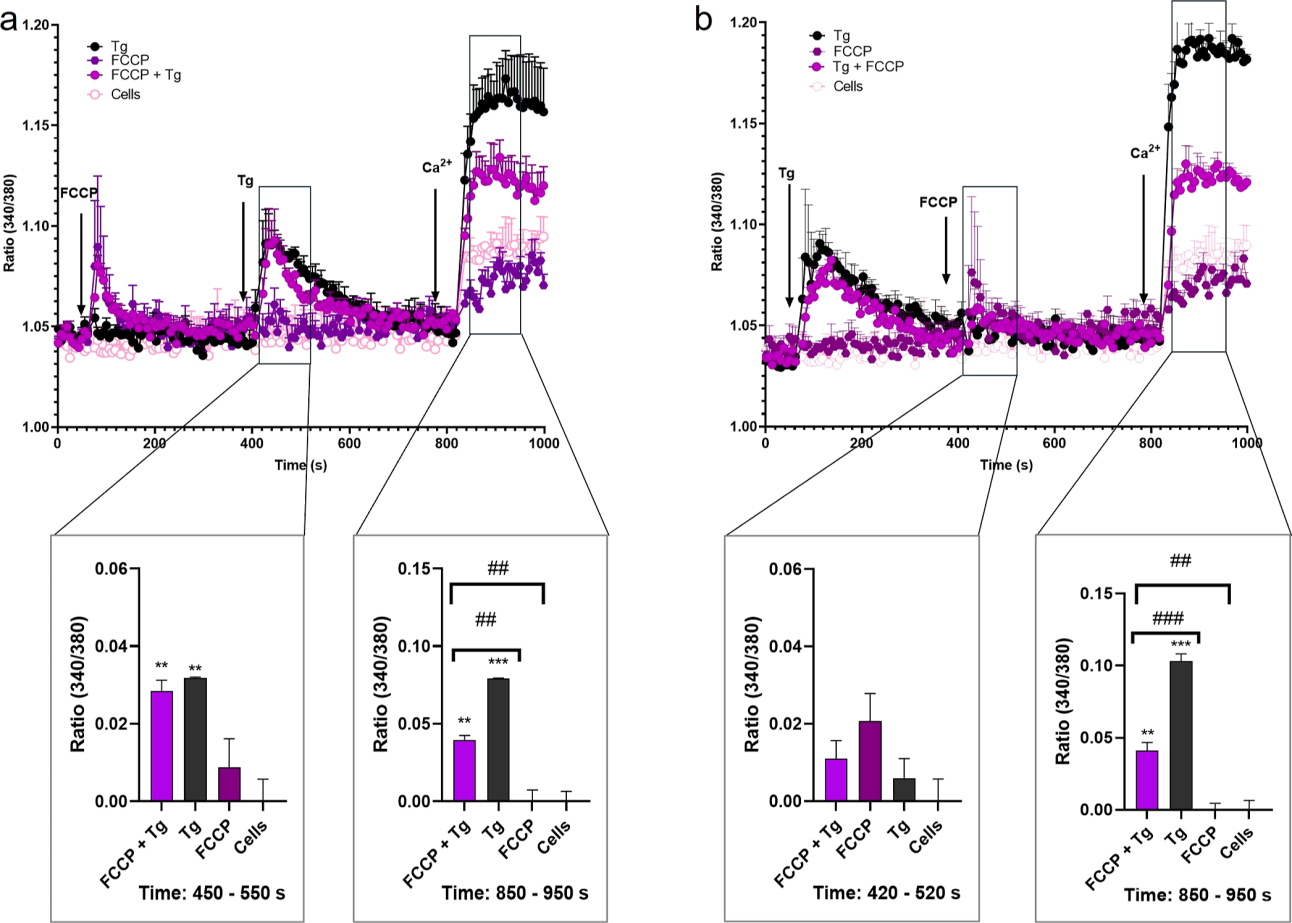
Effects of Tg and FCCP on the cytosolic Ca^2+^ profile of neuroblastoma cells. (a) Cytosolic Ca^2+^ profiles of SH-SY5Y cells pre-treated with 10 μM FCCP, followed by the incubation with 2 μM Tg. The left panel presents the quantification of Ca^2+^ release from intracellular pools (interval of seconds 450–550). The right panel represents the quantification of the Ca^2+^ uptake (seconds 850 to 950). (b) Cytosolic Ca^2+^ profiles of cells preincubated with 2 μM Tg, followed by the addition of 10 μM FCCP. The left panel shows the quantification of Ca^2+^ release at the seconds 420 to 520. The right panel presents the quantification of Ca^2+^ uptake (interval of seconds 850–950). Arrows indicate the addition of compounds and 1 mM Ca^2+^ to the bath medium. Statistical significance was determined by one-way ANOVA and Tukey’s test (***p ≤ 0.001, **p ≤ 0.01 compared to control cells; ###*p* ≤ 0.001, ##*p* ≤ 0.01, pairwise comparison). The mean ± SEM of three independent experiments carried out in duplicate.

Then, the mitochondrial uncoupler FCCP, which releases calcium from mitochondrial stores through the opening of the mPTP, was used in the presence of both ENNs to determine whether the functional state of the organelle altered the effect produced by the toxins on the cytosolic calcium profile.^21^ To this end, we first investigated how mitochondrial damage altered the Ca^2+^ profile of Tg, a compound with a known mechanism of action. Based on other assays performed by our research group, the concentration of 10 μM FCCP was chosen.^4,16^ FCCP and Tg and vice versa were sequentially added to the bath solution. In both treatments, the depletion of intracellular stores induced by FCCP or Tg, compared to that produced by them alone, remained unaffected (Figure 4a,b). However, the addition of 10 μM FCCP initially resulted in a reduction of 33.2% in Tg-induced Ca^2+^ uptake (Figure 4a). On the other hand, when FCCP was added after the SERCA inhibitor, calcium influx was reduced by 43.6% when the ion was added to the bath solution (Figure 4b).

**Figure 5:**
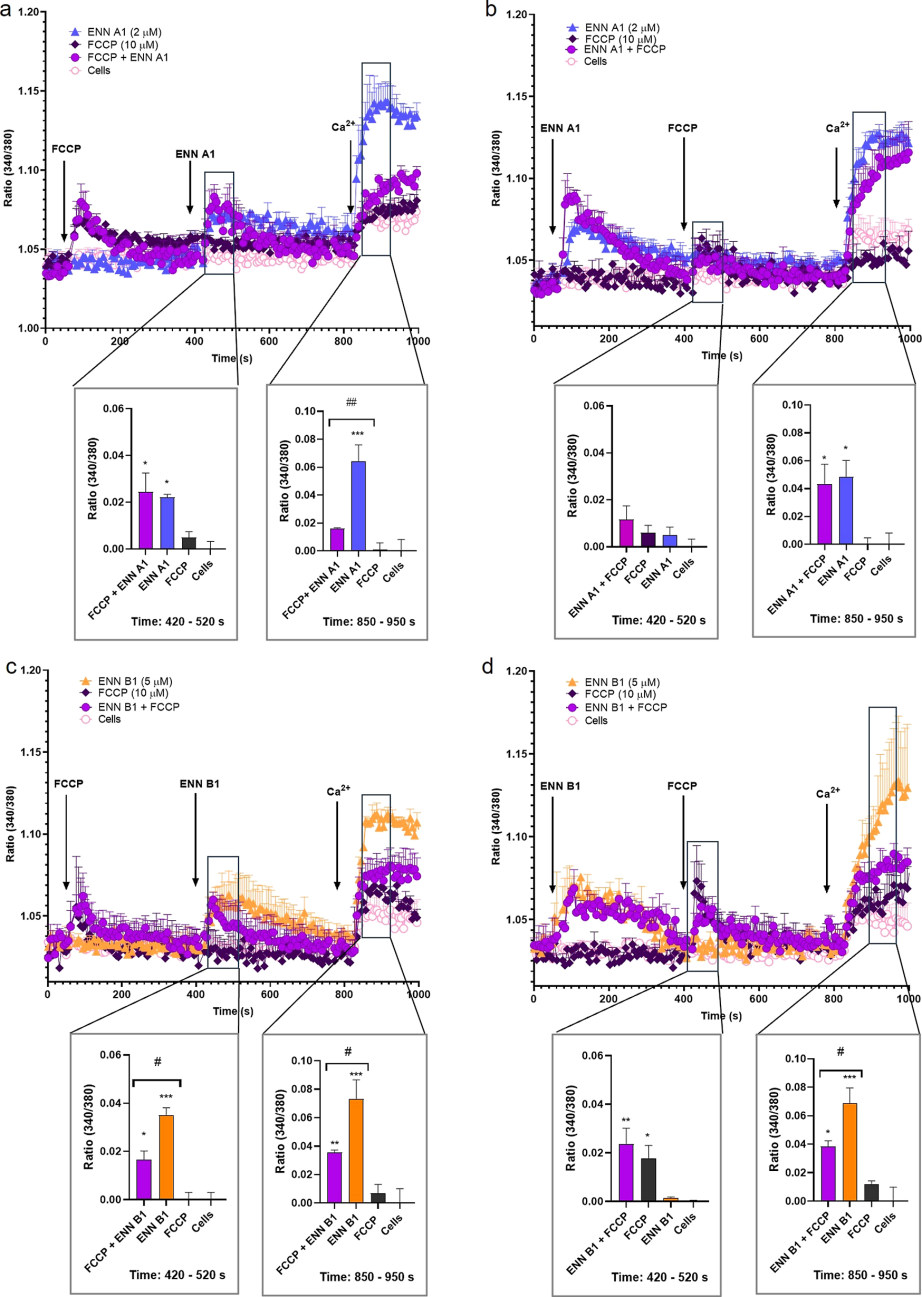
Effects of ENNs A1 and B1 and FCCP on the cytosolic Ca^2+^ profile of neuroblastoma cells. Cytosolic Ca^2+^ profiles of SH-SY5Y cells pre-treated with 10 μM FCCP, followed by the incubation with 2 μM ENN A1 (a), cells preincubated with 2 μM ENN A1, followed by the addition of 10 μM FCCP (b), SH-SY5Y cells treated with 10 μM FCCP and 5 μM ENN B1 (c), and cells preincubated with 5 μM ENN B1, followed by 10 μM FCCP (d). Arrows indicate the addition of compounds and 1 mM Ca^2+^ to the bath medium. The left panels show Ca^2+^ peak values between the seconds 420 and 520, and the right panels present the quantification of Ca^2+^ uptake values from the second 850 to 950. Statistical significance was determined by one-way ANOVA and Dunnett’s test (***p ≤ 0.001, **p ≤ 0.01, and *p ≤ 0.05 compared to control cells; #*p* ≤ 0.05, pairwise comparison). The mean ± SEM of three independent replicates carried out in duplicate.

Next, the effects of ENNs A1 and B1 on Ca^2+^ fluxes were determined in the presence of the mitochondrial uncoupler. In the case of ENN A1, preincubation with 10 μM FCCP did not affect the Ca^2+^ release from intracellular reservoirs, but the ion uptake was reduced by 51.6% (Figure 5a). When ENN A1 was added first, neither ion depletion from stores nor the Ca^2+^ uptake was altered (Figure 5b). For ENN B1, preincubation with FCCP reduced the toxin-induced ion depletion to about 58.0% and decreased the ion uptake by 38.8% (Figure 5c). When ENN B1 was added first, the effects of FCCP on the intracellular pools were not diminished. However, the ENN B1-induced ion entry was reduced by 38.8% (Figure 5d).

**Figure 6:**
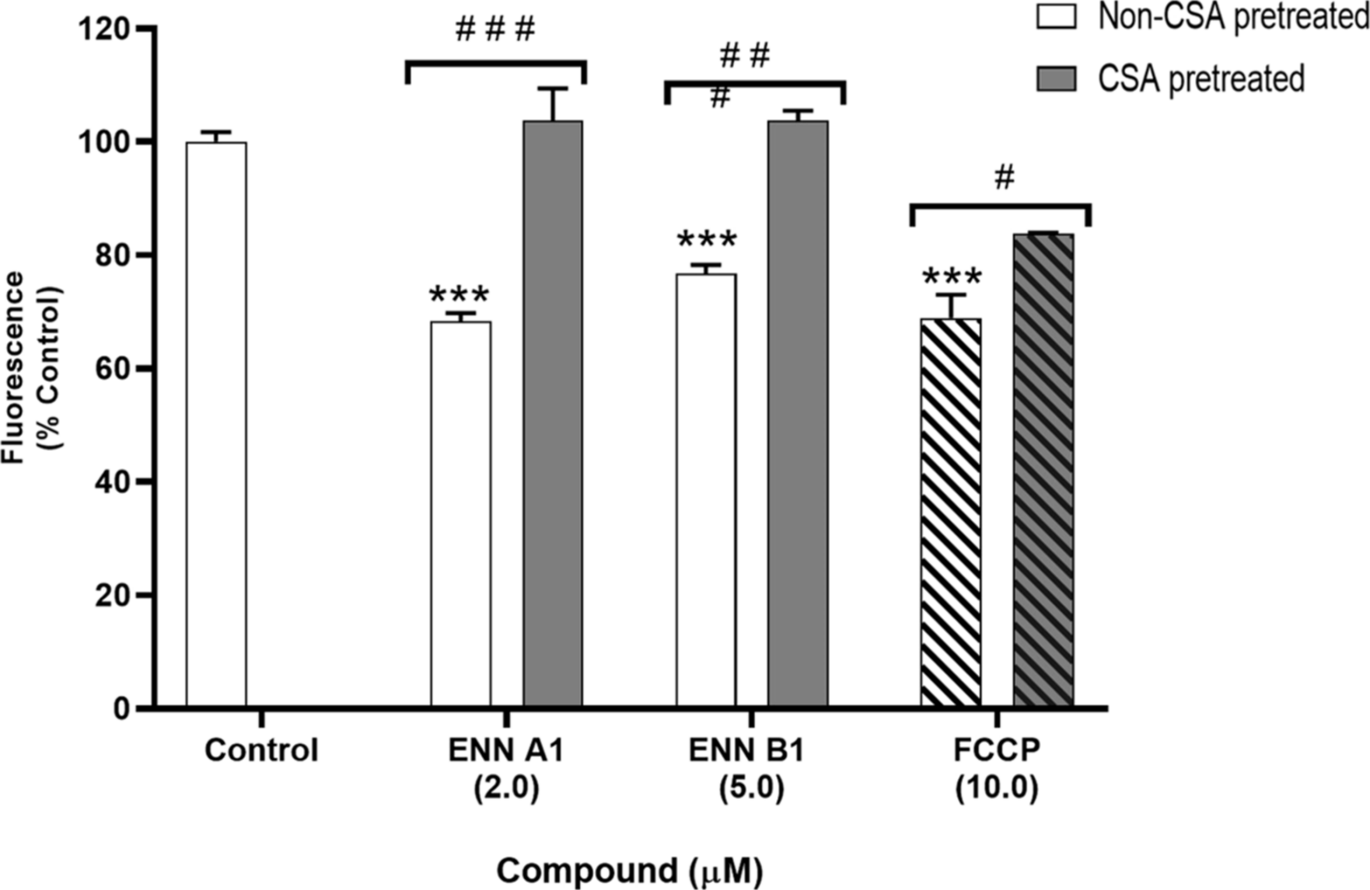
Effect of ENN A1 and ENN B1 on mPTP opening. SH-SY5Y cells were loaded with calcein-AM and CoCl_2_ and treated with ENNs for 10 min. The fluorescence of 10,000 events was analyzed by flow cytometry. FCCP (bars with angled black lines) at 10 μM was used as positive control, and CsA (gray bars) at 0.2 μM was added as an inhibitor of mPTP opening. The mean ± SEM of three independent experiments expressed as a percentage of control cells. Statical significance determined by one-way ANOVA and Dunnett’s test (***p ≤ 0.001, compared to control cells; ###*p* ≤ 0.001, #*p* ≤ 0.05, pairwise comparison).

As the mitochondrial damage induced by FCCP appeared to impact the effects of ENN A1 and ENN B1 on Ca^2+^ fluxes, it was investigated whether these toxins also affected organelle function by examining their effect on mPTP opening. With this objective, cells were stained with calcein-AM, which diffuses into the cytosol and accumulates in the mitochondria. Cytosolic fluorescence was quenched with CoCl_2_, which does not diffuse into mitochondria and allows one to monitor fluorescence changes due to mPTP opening.^22^ In this assay, 10 μM FCCP was used as a control to induce mPTP opening, and the pore inhibitor CsA was added to confirm the involvement of the channel in the mode of action of ENNs. It was observed that ENN A1 and ENN B1 reduced calcein fluorescence by 32.2 ± 1.0% (*p* < 0.001) and 23.1 ± 2.2% (*p* < 0.001), respectively, and that their effects were blocked with pretreatment with 0.2 μM CsA. Similar effects were observed with the mitochondrial uncoupler FCCP, which also inhibited the calcein signal by 27.8 ± 4.2% (*p* < 0.001), an effect reversed by preincubation with CsA (Figure 6).

## 4 Discussion

Although the cytotoxicity of ENNs A1 and B1 has been reported in different cell lines, the mode of action of these toxins has remained poorly explored. We had previously observed a proapoptotic effect of these compounds in human neuroblastoma cells, but the underlying mechanisms of this toxicity were only pointed out. Apoptosis appeared to be triggered by an increase in cytosolic calcium levels induced by toxins, as both led to a depletion of Ca^2+^ from intracellular stores and an increase in ion uptake. These observations suggested that their effects may be calcium-mediated.^4^ ENNs have been described as ionophores for many years, but it has been recently demonstrated that ENN A and ENN B are not Ca^2+^ ionophores.^6,9^ The current study also rejects the Ca^2+^ ionophoric behavior of ENNs A1 and B1 and highlights that, despite their structural similarities, the four analogues have different mechanisms of action, as it was suggested before.^3,4,9^

Regarding ENN A1, in our previous work, we proposed that its effect on calcium homeostasis could be mediated by the ER, as the toxin presented a Ca^2+^ profile similar to Tg^4^. In the present report, it was initially demonstrated that the toxin did not act as a calcium ionophore. First, the Ca^2+^ entry induced by the toxin was blocked with NiCl_2_, which obstructs calcium channels of the cell membrane, especially voltage-gated ones, by direct blocking of the pore.^18^ Subsequently, using SKF96365, a well-known SOC inhibitor, it was confirmed that the increase in the Ca^2+^ influx caused by the toxin was due to the activation of these channels. Then, the effects of ENN A1 were analyzed in the presence of FCCP, which uncouples the respiratory chain and is able to reduce Ca^2+^ entry through SOC in some cell models, such as SH-SY5Y cells.^16,23^ It was observed that the functional state of the mitochondria affects the Ca^2+^ influx induced by ENN A1 since preincubation with the uncoupler reduces calcium uptake through SOC. Under physiological conditions, the mitochondrion serves as a regulator of Ca^2+^, buffering its release into the cytosol from various pools and enabling the activation of SOC. Therefore, when FCCP disrupts the electron transport chain, the mitochondrion’s capacity to store calcium is reduced, thereby affecting the influx through SOC.^24,25^ In this sense, the effects of Tg on the induction of SOC-mediated Ca^2+^ entry were reduced when cells were treated with FCCP before and after the addition of the SERCA inhibitor. In the case of ENN A1, when the toxin has already acted, its effects on SOC entry are not reduced by FCCP. This effect could be due to the toxin acting on a component of the ER implicated in calcium efflux rather than on SERCA, which would be responsible for calcium entry into this organelle. When an inhibitor such as Tg blocks SERCA, it triggers the calcium exit pathways of the ER, leading to the activation of SOC entry and preventing the organelle from reabsorbing the ion. ENN A1 acts differently to Tg and also triggers SOC entry, suggesting that it may be involved in a calcium efflux mechanism from the ER. In this sense, inositol trisphosphate receptor (IP3R) and ryanodine receptor (RyR) are two channels involved in calcium release by ER that are expressed in human neuroblastoma cells.^26^ IP3R is closely associated with mitochondria as they form a complex with the chaperone glucose-regulated protein 75 (GRP75) and voltage-dependent anion-selective channel 1 (VDAC1), one of the components of mPTP. When Ca^2+^ is released through IP3Rs, it is transferred to the mitochondria via this complex. If the levels of the ion are too high, it can lead to mPTP opening and cell death.^27^ In this work, we have observed that ENN A1 induced the pore opening so that it could act as a modulator of the IP3R–CRP75-VDAC complex. In addition, IP3R has been described as the major reticular Ca^2+^ channel in the mitochondrial-ER contact sites, regions where the ER transfers Ca^2+^ to mitochondria.^28^ Therefore, IP3R could be a target of this toxin, which would explain why its effect on SOCs persists when administered prior to FCCP. Similar results were obtained with ENN A, which affects the ER and mitochondria, but the calcium profiles of the two toxins differ. These differences could be due to a distinct modulation of the IP3R-GRP75-VDAC1 complex by both analogues.^4,9^

The other analogue tested, ENN B1, was previously described to induce Ca^2+^ depletion from intracellular pools and ion entry in a dose-dependent manner.^4^ In the current study, we observed that preincubation with Tg reduced the ion depletion from intracellular compartments caused by the toxin, and addition of the toxin decreased the Tg-induced Ca^2+^ uptake. These results could also indicate an effect on the ER, as observed with ENN A1. Moreover, it was verified that ENN B1 was not an ionophore as NiCl_2_ decreased the ion uptake produced by the toxin. However, SKF96365 was unable to reduce the Ca^2+^ entry produced by the toxin, suggesting that ENN B1 activates a channel on the cell membrane other than SOC. Considering that Ca^2+^ release from the ER, either via IP3Rs or RyRs, triggers the entry of the ion via SOC, ENN B1 may be targeting another organelle, and the effects observed when preincubating with Tg may be due to interactions between different organelles, which could activate the RyRs in a secondary way.^29,30^

In this sense, the effects of ENN B1 on Ca^2+^ fluxes were also analyzed in the presence of the mitochondrial uncoupler FCCP. The addition of this compound was able to decrease the ENN B1-induced Ca^2+^ efflux from intracellular pools and the ion influx from the cell medium. However, when the toxin was added in the first place, no reduction in the mitochondrial emptying induced by FCCP was observed. Therefore, it appears that the mitochondrial status alters the effect of ENN B1 in calcium fluxes, but the toxin does not act directly in this organelle. These results are in agreement with previous studies that reported that ENN B1 did not affect MMP in either SH-SY5Y or HepG2 cells.^4,31^ Therefore, the observed effects of ENN B1 on mPTP opening might be secondary due to the massive calcium entry induced by the toxin rather than a direct effect on the organelle. Interestingly, its analogue, ENN B, with small structural differences, modulates calcium by directly targeting the mitochondria, endorsing the hypothesis that each ENN has a different mechanism of action.^9^

Although the ER and the mitochondrion are the most studied intracellular calcium reservoirs, there are other organelles with calcium storage capacity, such as the nucleus, Golgi apparatus, peroxisomes, or endolysosomes, but there is limited data on their role.^13^ A recent study in mouse embryonic fibroblasts suggests lysosomes as a possible early target of ENN B1, as the toxin seems to destabilize membrane-associated proteins, resulting in lysosomal alkalinization.^32^ Since lysosomal Ca^2+^ channels are sensitive to pH variations, the effects produced by ENN B1 on Ca^2+^ fluxes could be related to an interaction with these organelles. Moreover, lysosomes can be associated with the ER and mitochondria, which could explain the observed long-term effects on mPTP induced by ENN B1.^13,33^

In conclusion, we describe the different effects on the calcium homeostasis of ENNs A1 and B1 in human neuroblastoma cells. Contrary to previous assumptions, neither toxin acts as a calcium ionophore. The effect of ENN A1 on Ca^2+^ signaling involves a complex interplay with the ER and mitochondria that could activate SOC entry. On the other hand, ENN B1 affects Ca^2+^ depletion from intracellular stores via a pathway different from those organelles, possibly involving lysosomes. These findings provide an important starting point for understanding the different mechanisms of action of ENNs A1 and B1 and highlight the need for further research to fully unravel the intricate complexities of their effects within cellular calcium signaling pathways.

## Data Availability

All data used in this study are included in the manuscript figures.
